# A narrative review focusing on randomized clinical trials of vitamin D supplementation for COVID-19 disease

**DOI:** 10.3389/fnut.2024.1461485

**Published:** 2025-01-07

**Authors:** Limi Huang, Zhiwei Song, Chaosheng Lu, Shenwen Wang, Changsheng Guo, Xin-He Lai, Zhenfeng Zhao

**Affiliations:** ^1^Department of Pediatrics, The First Affiliated Hospital of Wenzhou Medical University, Wenzhou, Zhejiang, China; ^2^Department of Infection Diseases, Xianju County People's Hospital, Taizhou, Zhejiang, China; ^3^School of Information Engineering, Hebei GEO University, Shijiazhuang, Hebei, China; ^4^Shaoxing BWK Biotechnology Co., Ltd., Zhuji City High-Tech Entrepreneurship Center, Shaoxing, Zhejiang, China; ^5^Shenzhen Boya Gene Technology Co., Ltd., Shenzhen, China; ^6^Hebei Huiji Technology Co., Ltd., Shijiazhuang, Hebei, China

**Keywords:** randomized clinical trial, vitamin D, severe acute respiratory syndrome coronavirus 2 (SARS-CoV-2), COVID-19, serum 25-hydroxyvitamin D

## Abstract

Current evidence is inconsistent on whether vitamin D supplementation can prevent COVID-19 infection or improve its clinical outcomes. To better understand and look into the issue, we went through the background knowledge of COVID-19 and vitamin D, searched in Pubmed [by using key words in the title containing “randomized clinical trial”, “COVID-19”, and “vitamin D (25-hydroxyvitamin D, or cholecalciferol, or calcidiol, or calcifediol) supplementation”] for publications of studies on vitamin D/supplementation in COVID-19 patients, especially those about the randomized clinical trials (RCTs). After reviewing these papers, we did a short background review of vitamin D and the pathophysiology of COVID-19, summarized the key features of the 25 RCTs in text and tabulated in a table of some of the features, commented, compared and discussed the differences between RCTs (for example, change the serum 25-hydroxyvitamin D concentration from nmol/L to ng/mL, making the comparison easier). The take-home question of the review is that serum 25-hydroxyvitamin D concentration is an important indicator of the supplementation effect of vitamin D correction but may not be reliable in predicting the supplementation effect on the clinical outcomes of COVID-19.

## Vitamin D metabolism and function

**Figure 1:**
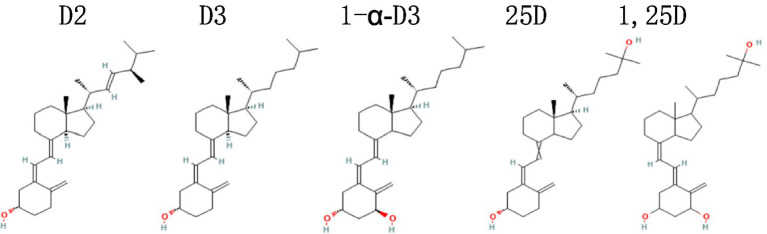
Maybe each of the 5 names above the 5 structures better in the midline of the corresponding structure. D2, vitamin D2, ergocalciferol; D3, vitamin D3, cholecalciferol; 1*α*-D3, 1-alpha-hydroxycholecalciferol, alfacalcidol; 25D, 25(OH)D, 25-hydroxycholecalciferol, calcidiol, calcifediol; 1,25D, 1,25-hydroxyvitamin D, 1,25(OH)2D, calcitriol.

Vitamin D (VD) obtained by the body mainly comes in two structurally different forms: vitamin D2 (D2, ergocalciferol; from dieting) and vitamin D3 (D3, cholecalciferol; from exposing skin to sun and dieting), with D2 having a methyl group in C24 and a double bond in C22–C23 (Figure 1). Once in the body, they are converted by enzymes into 25-hydroxyvitamin D (25D; calcidiol, calcifediol), an inactive form of VD (most often measured and used as the indicator of serum VD level), and then hydroxylated to form the biologically active 1,25-hydroxyvitamin D (1,25D; calcitriol) (1). Once activated, VD binds to the nuclear VD receptor (VDR) and forms a heterodimeric complex with retinoic acid X receptor that recognizes specific DNA sequences (VD responsive elements), resulting in expression of VD responsive genes via a variety of transcription factors. Particularly, 1,25D is a factor involving in regulating and promoting calcium absorption and intracellular transportation by increasing gene expression or concentration of the aforementioned proteins (2).

Approximately 3% of the human genome is under the control of 1,25D and regulated via the VD pathway (3). VDR is present in many human cells including various types of immune cells (such as dendritic cells, lymphocytes, macrophages, and monocytes), regulating the expression of a large number of target genes (~1,000) in these cells (4). Of the predicted 11,031 putative VDR target genes, more than 40% are assumed to involve in metabolism, about 20% in cell/tissue morphology, 10% each, respectively, in cell junction/adhesion, differentiation/development, and angiogenesis, and 5% with epithelial to mesenchymal transition (5).

Together, VD is a pleiotropic hormone and has profound impact on the human development, physiology, immunity through its connections to VDR.

## COVID-19 pathophysiology

Although the first COVID-19 pandemic is over, its pathophysiology is still not fully understood (6). For severe acute respiratory syndrome coronavirus 2 (SARS-CoV-2) to enter in the body and spread the infection, two sequential step reactions are required. The first step is SARS-CoV-2 invasion via host cell receptors (7), which requires S-protein priming to facilitate its entry of cells such as nasal, bronchial epithelial cells and pneumocytes (8). The second is cleavage of the spike protein by the transmembrane serine protease 2 (9).

Angiotensin-converting enzyme 2 (ACE2), one of the receptors for SARS-CoV-2, is most abundant in type II alveolar cells of the lungs, and also expressed in multiple tissue cells (airways, cornea, esophagus, ileum, colon, gallbladder, heart, kidney, liver, and testis) (10). ACE2 receptor-mediated SARS-CoV-2 infection initiates a cell signaling cascade, ultimately resulting in production of inflammatory cytokines, prothrombotic molecules, and acute phase reactants, which alone or together amplify the immune system’s responses which will protect or damage the surrounding tissues.

Upon their initial entry, SARS-CoV-2 may subsequently migrate from the nasal epithelium to the upper respiratory tract via the ciliated cells in the conducting airways (8), and start to proliferate, and people infected at this stage are highly infectious with high viral load but may remain asymptomatic (11). Viral transmission at the pre-symptomatic stage significantly contributed to the pandemic (12).

If the hosts are able to cope and mount a strong interferon-mediated response at this early stage, they may control the viral replication and limit the disease severity (13). Although the precise mediators of early viral clearance are not yet completely understood, a critical role of interferons (IFN) in viral elimination is related to their potent antiviral activity and robust upregulation in mild COVID-19, considering type I IFNs (IFN-*α*, −*β*, −*ω*) are indispensable in viral clearance (14). For patients failing to eradicate SARS-CoV-2 in its early stage, the disease may progress to the clinical phase or later stage of the infection, manifesting symptoms that may vary in severity and duration and resulting in a complex multisystem disorder (15).

Some proinflammatory cytokines are secreted following the binding and penetration of SARS-COV-2 into the respiratory epithelial cells (16). These cytokines include but not limited to: endothelial growth factor, granulocyte colony-stimulating factor (filgrastim), interferons (IFN-*γ*), chemokines (CCL2, CCL3, CCL5, CXCL8, CXCL9, and CXCL10) and interleukins (IL1*β*, IL4, IL6, IL8, and IL10), macrophage inflammatory protein A, monocyte chemoattractant protein 1, tumor necrosis factor-*α* (17). Simultaneous increase of multiple inflammatory cytokines at a certain stage forms the ‘cytokine storm’, making it difficult to pinpoint the specific mediator of inflammatory response (18). Higher levels of different cytokine profiles have been determined among severe SARS-COV-2 patients (14).

Together, COVID-19 is a viral infection but has broad impact far beyond the respiratory/pulmonary system, and can potentially affect or even damage other systems through the vast distribution of ACE2 receptor and the circulating molecules.

## VD deficiency/insufficiency is widespread

According to the Endocrine Society’s Practice Guidelines (19) and “Vitamin D deficiency 2.0: an update on the current status worldwide” (20), 25D level at <20 ng/mL (50 nmol/L; according to https://unitslab.com/node/84#google_vignette) is VD deficiency, 21–29 ng/mL (51–74 nmol/L) VD insufficiency, and ≥ 30 ng/mL (75–250 nmol/L) VD sufficiency. Of note that this criterion is for the consideration of maximum musculoskeletal health.

One study estimated that at a given time the VD concentration is suboptimal in half of the world’s population across all age groups and residing in both developed and developing countries (21, 22). It is also estimated that globally there were more than one billion VD deficient people (23), illustrating that VD insufficiency/deficiency is a worldwide public health problem. For example, almost 25% of the subjects in USA had vitamin D deficiency (24), and 34.76% of a total of 227,758 participants in South America had vitamin D deficiency (25).

## Interplay between VD and COVID-19

People, young and old, have witnessed and experienced the huge impact of the COVID-19 pandemic on our psychological/physical health and everyday life. Considering the wide distribution of VDR in and the profound impact of COVID-19 on the human body, the interplay between VD and COVID-19 might be far more complicated than what people have so far learned, and the exact pathways and mechanisms of their interplay are so far difficult to pinpoint. On the one hand, SARS-CoV-2 infection can cause an inflammation status leading to VD deficiency (26); on the other hand, VD deficiency might be a risk factor for COVID-19.

Soon after the emergence of COVID-19 at the end of 2019, a few researchers had suggested the connection of low VD with COVID-19, and some even hypothesized to use VD supplementation as an adjuvant therapy, for prophylaxis purpose to reduce COVID-19 severity, or even for a trial in COVID-19 patients (27, 28).

To better understand the background of VD-COVID-19 studies, about two dozens of work were also herein reviewed and the main results were summarized (Supplementary Table S1). Around ten studies show that VD insufficiency/deficiency was significantly related to the infection, severity, and mortality of COVID-19, in contrast to a few failing to show the connection. About equal number of studies either show or deny that VD supplementation helped in reducing ICU admission rate or mortality (Supplementary Table S1). A recent work supports the notion that VD deficiency (25D < 12 ng/mL) is an independent biomarker weathering the worsening of COVID-19, particularly in hospitalized non-severe patients (29).

Comparing to patients recovering without long COVID, those long COVID patients were found to have lower 25D levels (30). COVID-19 patients tend to have high prevalence of hypocalcemia (31), and daily D3 supplementation (either 2000 IU or 10,000 IU) for 2 weeks was able to increase the serum calcium level (32).

The first case study using VD supplementation in COVID-19 patients was conceived soon after the outbreak, started in April 2020, and finished half year later. Four VD insufficient/deficient patients diagnosed with COVID-19 were given with either daily D3 (1,000 IU, standard dose) or D2 (50,000 IU, high dose) for 5 days (33). The 25D baselines of all four patients were < 22 ng/mL, bordering between insufficient/deficient level. On day 6, the serum 25D of the two patients receiving high dose D2 reached 39.9 ng/mL and 50.5 ng/mL respectively, contrasting to the minimally changed level of the other two receiving standard dose D3.

The results of the first of the 25 reviewed RCTs were published in August 2020 (discussed below), and the past 4 years brought in more results from more studies of different natures (larger scale, randomized, single or double blinded, with control/placebo group), with RCTs being the main focus of this review.

## Key features of the 25 randomized clinical trials (RCTs #1–25) of vitamin D supplementation for COVID-19 disease

To make the manuscript concise, the following abbreviations for various types of vitamin D will be used: VD, vitamin D; D3, vitamin D3, cholecalciferol; ERC, extended-release calcifediol; 1α-D3, 1-alpha-hydroxycholecalciferol, alfacalcidol; 25D, 25(OH)D, 25-hydroxycholecalciferol, calcidiol, calcifediol; 1,25D, 1,25-hydroxyvitamin D, 1,25(OH)_2_D, calcitriol.

For similar reasons, the following abbreviations are used below: ARDS, acute respiratory distress syndrome; BMI, body mass index; CRP, C-reactive protein; CT, computed tomography; ICU, intensive care unit; CLIA, chemiluminescent immunoassay; d, day(s); ELIA, electrochemiluminescence immunoassay; h, hour; HPLC, high-performance liquid chromatography; LC-(T)MS, liquid chromatography (tandem) mass spectrometry; PaO2/FIO2, partial pressure to fractional inspired oxygen; rRT-PCR, real-time reverse transcription polymerase chain reaction; rSOFA, respiratory Sepsis related Organ Failure Assessment; WHO, World Health Organization; y, year(s).

#1 (34): 76 Spanish hospitalized patients (mean age: 53 y) were enrolled in the ‘Pilot Covidiol’ study (NCT04366908). Their COVID-19 infection diagnoses were made by radiographic patterns of viral pneumonia and by positive SARS-CoV-2 PCR. The sample size was calculated based on certain assumptions. The 25D serum levels at enrollment or after VD supplementation were not reported. The control group had a higher percentage of hypertension at enrollment.

All participants had ‘standard’ of COVID-19 treatment (the best therapy available at that time per hospital protocol, a combination of hydroxychloroquine and azithromycin). The VD group (50 patients) received the first oral dose (0.532 mg) of calcifediol on the day of admission, followed with 0.266 mg on d 3 and d 7, and then weekly until discharge or ICU admission (calcifediol is 25D; according to https://vitamored.com/products/vitamored-vegan-vitamind3-calcifediol, 0.532 mg of calcifediol = 106,400 IU of D3). The rate of ICU admission and deaths were the prespecified outcomes of effectiveness of treatment. When compared to those without 25D addition (50%, 13 in total), the need for ICU admission of the 25D-supplemented patients (2%) was significantly reduced.

#2 (35): A small group (*n* = 40) of asymptomatic or only mildly symptomatic COVID-19 patients without comorbidities were enrolled in the SHADE study in India (NCT04459247), 14 of them were randomized to receive placebo (5 mL distilled water), and 16 to receive daily 60,000 IU of cholecalciferol (D3; oral nano-liquid droplets) from the first day of enrollment until their serum 25D reached the goal (>50 ng/mL) by d 14. Although only 40, the sample size was well calculated. For those reaching the goal earlier by d 7, they continued to receive 60,000 IU at d 14. The proportions of participants who turn SARS-CoV-2 negative (confirmed twice at 24 h interval) before week 3 was set as the primary outcome, and the other outcome was the change in the level of inflammatory markers after treatment. At d 0 and 7, 25D levels were assessed by ELIAusing a supplied kit. Oro-pharyngeal swabs were obtained at six time points (days 5, 7, 10, 14, 18, 21) and SARS-CoV-2 RNA detection was performed by RT-PCR.

The serum 25D level in ten out of the 16 patients achieved >50 ng/mL by d 7 and another two by d 14; 2 weeks of D3 supplementation raised the median serum 25D from 8.6 ng/mL to 51.7 ng/mL (*p* < 0.001). Greater proportion of the VD supplemented patients turned their viral RNA tests negative (62.5% vs. 20.8% in control group, *p* < 0.018), and with a significant decrease in fibrinogen (*p* = 0.007).

#3 (36): 69 mild to moderate COVID-19 hospitalized patients (20–75 y) who were newly diagnosed (no more than 3 d by RT-PCT) were enrolled in a trial from 29 July–22 September 2020 in Saudi Arabia to orally take either a high (5,000 IU, *n* = 36) or low (1,000 IU, *n* = 33) daily D3 supplementation for 2 weeks. A mild–moderate COVID-19 case was defined by the Saudi Ministry of Health that the patient on presentation had clinical symptoms and required supportive care but not oxygen. Serum 25D was assessed using the CDC-approved CLIA assay as certified by the VD Standardization-Certification Program (VDSCP); Of note, the VD baseline of 40 cases (55%) was within the deficiency level.

Only the 5,000 IU treatment significantly increased 25D levels (baseline 53.4 nmol/L = 21.36 ng/mL vs. after supplementation 62.5 nmol/L = 25 ng/mL; *p* = 0.001 without adjustment, and *p* = 0.003 after adjustment for covariates: age, sex, baseline BMI, and D-dimer). Only the high dose (but not 1,000 IU) D3 supplementation shortened the time to recovery from cough and gustatory sensory loss, with caveats that the 1,000 IU group (25D level: baseline 63 nmol/L = 25.2 ng/mL vs. after supplementation 59.9 nmol/L = 23.96 ng/mL) had significantly higher BMI (*p* = 0.02) at enrollment and significantly older (*p* = 0.03). A significant increase in neutrophil (*p* = 0.03) and urea (*p* < 0.001) was noticed in the high dose group after supplementation.

#4 (37): 321 PCR-negative Mexican healthcare workers highly exposed to COVID-19 prior to vaccination were enrolled between July 15 to December 30 of 2020, and half of them were randomly assigned either to receive D3 or placebo (capsules with identical appearance containing 450 mg cornstarch). Sample size (156 subjects per group) was calculated based on a binary result, and randomization was done by using a software (Research Randomizer; https://www.randomizer.org/). The placebo group was older and had a higher frequency of diabetes than the supplementation group. Serum 25D and antibody tests were measured at baseline and at d 45, using a Waters ACQUITYH UPLC Class coupled to a Xevo TQD, with an APCI Ion SABRE II probe and a solid phase extraction cartridge. Chromatographic analyses were performed with a C18-column at 50°C. The prespecified primary outcomes were the rate of SARS-CoV-2 infection by RT-PCR tests and the severity of the disease. Secondary endpoints were set as the reduction of VD deficiency prevalence and the frequency of treatment-associated adverse events.

Regardless of the baseline level (mean 18.3 ng/mL), D3 supplementation (4,000 IU daily for 30 d) significantly increased the serum 25D concentration at d 45 and lowered the COVID-19 infection rate (6.4% D3 group vs. 24.5% placebo group, *p* < 0.001) in the follow-up period (days 7, 14, 21, 28 and 45), after adjusting for a few factors (age, comorbidities, vitamin D deficiency at baseline, as well as by site of study and type of personnel). By multivariate analysis, the authors could predict COVID-19 risk through Delta serum 25D concentration.

#5 (38): A multicenter trial (NCT04344041) enrolled 254 French COVID-19 patients (median age: 88 y) between April 15 and December 17 of 2020, and within 72 h after the diagnosis gave some of them (the intervention group) orally a single D3 treatment on the day of inclusion administered under medical supervision (ideally during food intakes for better absorption) to compare the 14 d overall survival between the high (400,000 IU) and standard dose (50,000 IU) groups. Patients positive in RT-PCR test and/or chest CT scan were allocated by dynamic randomization using a minimization algorithm and considering 6 criteria. All patients had at least one of COVID-19 worsening risk factors (age ≥ 75 y, SpO2 ≤ 94%, or PaO_2_/FiO_2_ ≤ 300 mm Hg). 14 d overall mortality was the prespecified primary outcome, calculated after adjusting for randomization strata (age, delirium, hospitalization, ongoing cancers, oxygen requirement, profuse diarrhea, sex, and use of medications). Secondary outcomes were between-group comparison of safety, overall mortality within 28 d after enrollment, and mortality at both time points (14 and 28 d). Three follow-up visits (at 7, 14, and 28 d) were scheduled after the randomization. Blood samples from baseline (before D3 administration) and d 7 (±1 d) were obtained in the morning. Serums from thawed samples (within 4 h) were analyzed locally at each site to measure changes in serum 25D concentration by CLIA. Immunoassay kits recognize both D2 and D3. No participants were in the ICU at the time of entering the trial.

The difference of the serum 25D concentration at baseline was not significant between groups (high dose 53.0 nmol/L = 25.2 ng/mL vs. standard dose 43.0 nmol/L = 21.36 ng/mL), but the serum 25D level of the high dose group after week long supplementation was significantly higher (*p* < 0.001) than the standard dose group (150.5 nmol/L = 60.2 ng/mL vs. 64.5 nmol/L = 25.8 ng/mL). Compared to the standard dose, the high dose treatment reduced the overall mortality at d 14 (unadjusted *p* = 0.20; adjusted *p* = 0.049) but not after 28 d. The weakness of absence of placebo group was partly compensated by controlling for imbalances of randomization strata and prognostic factor baselines. Even though the 25D level (150.5 nmol/L = 60.2 ng/mL) after 400,000 IU D3 intervention was relatively high, the protocol-specified adverse events of interest (37 items) were not significantly different between the two groups. Notably, corticosteroids were prescribed for 34 participants (27%) in the high-dose group and 41 (32%) in the standard-dose group.

#6 (39): Belgian (Caucasian, VD deficiency defined as serum 25D concentration ≤ 20 ng/mL and hospitalized for confirmed SARS-CoV-2 infection at screening) COVID-19 patients of unspecified severity were enrolled in the trial (NCT04636086) from August 2020 to August 2021. Participant sample size was not formally calculated. The trial lasted for a maximum of 9 weeks (up to 6-week treatment period and a maximum of 3-week follow-up period). The severity of the disease was assessed by the ordinal WHO scale for clinical improvement both at randomization and efficacy evaluation. The study treatment was under the supervision of the clinical staff, leading to 100% compliance. The last day of the 6-week treatment period was the last day of hospitalization or d 36, whichever was first. Prespecified outcomes of effectiveness included 25D serum level, ordinal scale for clinical improvement as recommended by the WHO, hospitalization length, intensive care unit admission, time until absence of fever, need for supplemental oxygen, non-invasive ventilation, high-flow oxygen devices, invasive mechanical ventilation or additional organ support and death. The Fujirebio 25-OH VD assay on Lumipulse G1200 analyzer was used to screen the 25D concentrations, which showed excellent concordance with the LC–MS/MS method used in the laboratory and provided results in a fast turnaround time, fitting the needs of a screening. To rapidly restore the D3 level, 4 consecutive daily VD doses of 25,000 IU were first given to patients of the intervention group, and 25,000 IU per week (up to 6 weeks) to maintain the 25D level. To assure a standard VD supplementation to those with possibly more severe VD deficiency, all ICU patients with enteral nutrition would additionally receive 600 IU VD per day.

D3 supplementation increased their serum 25D level from below 20 ng/mL to 29.9 ng/mL at the end of the study, improved the clinical outcome (clinical recovery, hospitalization length, supplemental oxygen duration). The median length of hospital stay significantly decreased in the VD group compared to the placebo group (4 d for the VD group vs. 8 d for the placebo group; *p* = 0.003); and none of the patients treated with VD were hospitalized after 21 d compared to 14% of the patients treated with placebo. Among all the patients who needed supplemental conventional oxygen, the administration of VD significantly decreased the duration of treatment (4 d vs. 7 d; *p* = 0.012). At d 7, 71% of the patients supplemented with VD switched from the moderate to the mild category of the scale compared to 18% in the placebo group (*p* = 0.0048). At d 36, 90% of the patients from the VD group were no more infected compared to 77% in the placebo group. There was no effect of age, arterial hypertension, BMI, cardiac pathology, diabetes, gender, height, hepatic failure, vaccinal status and weight on the primary endpoint (*p* > 0.05).

#7 (40): 45 moderate COVID-19 Mexican children (81% had some comorbidity, nearly half were obese) who required hospitalization and supplemental oxygen were enrolled in the trial (NCT04502667) and their disease severity was accordingly classified as mild, moderate, severe or critical. Patients were randomly assigned by a researcher to the VD supplementation group or to the control group not receiving VD. Supplementation started on the day of enrollment by receiving a daily 1,000 IU D3 for children <1 y or 2000 IU for those 1 to 17 y old, and continued during hospitalization for a minimum of 7 d and a maximum of 14 d. The prespecified outcome variables were progression of oxygen requirement, development of complications, and death.

The trial measured the baseline serum 25D levels (median, 13.8 ng/mL in the VD group and 11.4 ng/mL in the control group) using the Fujirebio 25-OH VD assay on Lumipulse G1200 analyzer but without the endline data. The trial was designed to last from 24 March 2020 to 31 March 2021 but stopped prematurely after seeing that none of the basal VD values of the patients were at normal levels, and for ethical reasons decided to supplement VD to all hospitalized COVID-19 patients. The original outcomes were set to measure the progression of oxygen requirement, the development of complications, and death.

#8 (41): After power calculation for sample size, 116 Egyptian patients (mean age ≈66 y) hospitalized with pneumonia (verified by chest CT scan, a positive COVID-19 RT-PCR and a hyperinflammation status) were enrolled in the trial (NCT04738760; From Dec. 2020 to June 2021) and allocated using a table of random numbers to receive either low (oral 1 mcg of 25D/d for five d; 1 mcg 25D equals to 200 IU D3 as per https://vitamored.com/products/vitamored-vegan-vitamind3-calcifediol) or a single high dose D3 treatment (200,000 IU intramuscularly). Serum 25D levels were not reported. D614G mutant strain was detected in patient samples, which was prevalent in late 2020. The following treatment was also given to all patients per day, for at least five d: 25 mg quetiapine (bedtime), 4 g paracetamol (1 g every 6 h), 6 mg dexamethasone, 400 mg hydroxychloroquine, 400/100 mg lopinavir/ritonavir twice, or 200 mg remdesivir loading dose followed by 100 mg.

The prespecified primary outcome was set as improvement of oxygenation parameters. Numerous secondary outcomes were set, including: hospital stay length, mortality, variation in inflammatory markers (CRP, ferritin, and lactate dehydrogenase), and occurrence of secondary infections and adverse event.

The single D3 high dose was found to be associated with better clinical improvement (clinical improvement, length of hospital stay, need for high oxygen, need for a mechanical ventilator or non-invasive mechanical ventilator, the occurrence of sepsis) and fewer adverse outcomes compared to low-dose VD group. Compared to the low-dose group, fewer patients in the high-dose VD group needed an invasive mechanical ventilator (*p* = 0.03), required ICU admission (*p* = 0.016), showed secondary bacterial infection in the form of sepsis (*p* = 0.04), had a decrease in basal CRP value (*p* = 0.007), and more patients showed clinical improvement (*p* = 0.03).

#9 (42): 134 mild to moderate COVID-19 patients in USA were enrolled in the REsCue trial (NCT04551911) to take ERC (extended-release calcifediol, with a lipophilic fill gradually releasing 25D) by an initial big dose (300 mcg on d 1–3; 300 mcg equals to 60,000 IU as per https://vitamored.com/products/vitamored-vegan-vitamind3-calcifediol) and follow by a maintenance dose (60 mcg on d 4–27; 60 mcg equals to 12,000 IU of D3). Participants were recommended to remain fasting for 3 h after dosing. Serum total 25D was analyzed by LC–MS, and total 1,25D by CLIA.

Thirty-four symptoms were self-reported daily using the FLU-PRO Plus questionnaire (an outcome tool validated for respiratory tract viral infections), positive for SARS-CoV-2 within the previous 3 d via RT-PCR or substitutable FDA-authorized test; mild to moderate COVID-19, defined as the absence of clinical signs indicative of more severe disease such as oxygen saturation < 94% or respiration rate > 30 breaths per minute. The two specified primary end points were attainment of the targeted serum 25D level by d 14, and time to resolution of five composite COVID-19 symptoms (trouble breathing, chest congestion, body aches or pains, chills or shivering, dry or hacking cough) which were part of the chest/respiratory and body/systemic domains of the questionnaire for which mean scores of ≥1.5 were required for enrollment. Secondary end points included time to resolution of each composite symptom and of aggregated symptoms as a function of serum 25D.

This dosing strategy raised serum 25D from 37 ng/mL to 82 ng/mL (*p* < 0.0001) by d 7 and remained elevated to the end of the study (d 28), and accelerated the resolution of respiratory symptoms and mitigated the risk for pneumonia. In the full analysis set (FAS), 81% of patients in the ERC group achieved 25D levels of ≥50 ng/mL versus 15% in the placebo group (*p* < 0.0001), respiratory symptoms resolved 4 d faster when 25D was elevated above baseline level at both d 7 and 14.

#10 (43): Supplementation with alfacalcidol (1*α*-25D, a synthetic analogue of 25D) in addition to standard care for COVID-19 was applied to Thai COVID-19 pneumonia patients (≥ 18 years) in the trial (TCTR20210906005) between July 2020 and March 2022, with the supplementation starting on the day of enrollment until the end of hospitalization. Of note, some patients were diagnosed with pneumonia either on admission or developed pneumonia later. Among the 241 patients, 43.57% were VD insufficiency (105 patients), 26.56% VD deficiency (64 patients), and 5.39% severe VD deficiency (13 patients). All patients received antiviral therapy, and over half of the participants used corticosteroids [i.e., 76/147 patients (51.70%) in the control group and 82/147 patients (55.78%) in the intervention group].

The prespecified clinical outcomes were set as: pneumonia treatment duration, length of hospital stay, and change in pneumonia severity index between enrollment and discharge. The secondary outcomes were subgroup analyses according to the need for supplemental oxygen, 25D concentration (< 12 and < 20 ng/mL), prednisolone administration (≥ 1 mg/kg/d), lymphopenia (absolute lymphocyte count, < 1,000 cells/mm^3^), and CRP concentration (< 30, ≥ 30, ≥ 40, and ≥ 50 mg/L).

It is odd the authors reported the baseline 25D level at enrollment (supplementation group 22.50 ng/mL vs. placebo group 20.83 ng/mL, both within the insufficiency range 20–29.99 ng/mL) but not the level after supplementation. 1*α*-25D supplementation was not beneficial to all patients, only benefited those who required supplemental oxygen or received high-dose corticosteroid therapy or had high CRP concentrations (> 30 mg/L) at the time of treatment initiation.

#11 (44): In this international, multicenter trial (ACTRN12620000557932) carried out between Jan. and June 2021, D3 (5,000 IU daily for 14 d) was given as a combo (together with hydroxychloroquine, azithromycin, zinc, with/without vitamin C) to COVID-19 hospitalized patients. COVID-19 patients were first diagnosed at the time of enrolment by PCR testing via nasal and/or oral swab. 73% of the patients had comorbidities, ranging from diabetes (35%), heart disease (36%) to lung disease (34%). None of the patients had optimal VD levels (≥75 nmol/L = ≥30 ng/L); specifically, 55% of them were severely deficient (<25 nmol/L = <10 ng/L), 42% deficient (<50 nmol/L = <20 ng/L), and only 3% at insufficient level (<75 nmol/L = <30 ng/L). Serum 25D levels after supplementation were not reported. D3 was not controlled (i.e., no D3-alone group), therefore the role of D3 could not be certain. The primary outcome was mortality or need for invasive mechanical ventilation within the first 15 d from enrolment, and the secondary outcome includes the WHO Master Protocol ordinal score at d 15.

Nevertheless, the study concluded that the combo protocol was safe and effective in treating COVID-19 infection, and VD deficiency to be a high-risk factor of severe COVID-19 disease and hospitalization. The lower the vitamin D level, the higher the probability of being admitted to the ICU (14.2 nmol/L = 5.7 ng/mL vs. 25.1 nmol/L = 10 ng/mL, *p* < 0.0001). Furthermore, a statistically significant correlation was found between lower baseline VD levels and longer hospital stay (*p* = 0.003).

#12 (45): 50 mild to moderate (not yet so severely-ill to require hospital admission) and RT-PCR confirmed COVID-19 patients were enrolled in Pakistan between 2 Sep. 2021 and 28 Nov. 2021, and randomization was carried out using computer-generated random number tables. Among other outcomes for this pilot study, the primary outcome was set as patient testing negative for SARS-CoV-2 in the RT-PCR analysis, and the secondary outcomes included improvement in the COVID-19-associated acute symptoms, and laboratory biochemistry.

Compared to the control group using standard of care only (including paracetamol with or without azithromycin), the combo (co-supplementation of 360 IU D3 together with curcumin and quercetin) cleared the SARS-CoV-2 viral infection faster and relieved the acute symptoms quicker at the end of supplementation for 14 d, probably by modulation of the early-stage hyperinflammatory response. Serum 25D levels were not reported, and no definite role of D3 could be certain since no D3 alone group was designed in the trial (NCT05130671).

#13 (46): 120 mild to moderate COVID-19 patients tested positive in SARS2-CoV-2 PCR were enrolled in the trial (NCT04981743) after the sample size was calculated (GPower v.3.1.9.4). D3 (2,000 IU) was given to the D3-alone group and to the *Nigella sativa-*D3 combination group in addition to the standard therapy to see the supplementation effect on the clinical outcome. Serum 25D levels were not measured. The main outcomes of this study were the viral clearance judged by a negative PCR test result and the symptom alleviation during the duration of 14 d. Patients tested negative on d 7 were considered having cleared of the virus. Negative COVID-19 results by PCR test were recorded on the 7th and 14th d of therapy.

The *Nigella sativa-*D3 combination group was superior compared to those of the other studied arms, and the independent contribution of D3 supplementation could not be certain. Comparing to the control group, the other groups had reduced severity of cough, diarrhea, fatigue, and pharyngitis. However, the four groups showed non-significant relief of symptoms (ageusia, anosmia, headache, rhinorrhea, shortness of breath, and vomiting). VD3 group showed an increase in lymphocyte count (137/μL) and total leukocyte count (1.17 × 10^3^/μL) at the end of the study period.

#14 (47): 181 COVID-19 Indian patients were enrolled in the trial (NCT04641195) from April 2021 and ended in Feb. 2022 after the sample size was calculated using methodology for survival times, and randomization was done by an independent statistician. Infection was confirmed by rapid antigen test or RT-PCR.

The prespecified primary outcome was set as the time to resolve cough, fever, and shortness of breath. The secondary outcomes included: duration of individual symptoms and hospital stay; need for assisted ventilation; all-cause mortality; and blood biomarkers (nutritional, inflammatory, and immunological markers). Hospital staff oversaw inpatient participants taking their daily supplements or reminded those who left the hospital to take their supplements during regular telephone follow-ups (completed in August 2022). Participants were followed either daily in person if in hospital or every 3 d via telephone (upon leaving the hospital) for 8 week to collect data on COVID-19 symptoms, supplement compliance, and any adverse events.

D3 supplementation (180,000 IU bolus at enrollment, then 2000 IU daily from the 2nd d for 8 weeks) did not improve COVID-19 treatment outcomes (resolution of cough, fever, and shortness of breath).

#15 (48): The CORONAVIT program (NCT04579640) lasted more than one y (from 1 May 2020 to 6 Oct. 2021), enrolled 6,200 cohort participants (about 95% were white people, and about 90% resided in England), the largest scale among the 25 RCTs, after the sample size was calculated on certain assumptions and by using a graphical user interphase, and randomization was done by using a computer program (Stata v14.2). For 6 month, it delivered daily either low (800 IU, *n* = 1,550) or high (3,200 IU, *n* = 1,550) of D3 to UK residents (≥16 y) whose blood 25D concentrations were under 75 nmol/L (30 ng/mL).

The primary outcome was the proportion of participants with acute respiratory tract infection of any cause confirmed by a doctor or a swab test. Secondary outcome was the proportion of COVID-19 participants confirmed by a swab test.

25D concentrations were measured using a blood spot testing kit testing the capillary blood, and concentrations of 25D3 and 25D2 were determined in dried blood spot eluates using LC-TMS after derivatisation and liquid–liquid extraction [good overall agreement was observed between using the blood spot method and plasma 25D concentrations in paired capillary and venous samples, showing a minimal overall bias of −0.2% (bias range − 16.9–26.7%)].

2,674 (86.3%) of the intevention group had baseline 25D concentrations <75 nmol/L (<30 ng/mL, defined as deficient or suboptimal). Compared to baseline level (66.6 nmol/L = 26.64 ng/mL), both supplementation strategies significantly increased the mean 25D concentrations (low dose group 79.4 nmol/L = 31.76 ng/mL, high dose group 102.9 nmol/L = 41.16 ng/mL) measured after 6 month VD supplementation but none reduced the risk of COVID-19. The incidence or severity of acute COVID-19 or prolonged symptoms were not statistically and significantly different between the low or the high dose group compared with the no supplementation group.

#16 (48): 120 Brazilian patients (over half were white, 30% mixed ethnicity, 10% black) with moderate to severe COVID-19 infections were enrolled from June 2 to August 27 of 2020 in the trial (NCT04449718) to receive a bolus single dose of D3 (200,000 IU). The mean time from the onset of symptoms to randomization was 10.3 d, and from hospitalization to randomization was 1.4 d. At the time of enrollment and at some point during the hospital stay, patients had their COVID-19 diagnoses confirmed by PCR testing or ELISA to detect IgG against SARS-CoV-2. Overall, 125 of 210 patients (59.5%) had CT scan findings suggestive of COVID-19 and 147 of 237 (62.0%) had a PCR test result positive for SARS-CoV-2.

Quantitative outcomes were assessed at baseline when enrolled and compared to those when discharged. 212 (89.5%) required supplemental oxygen at baseline. The primary outcome was set as the length of hospital stay; and the secondary outcomes were prespecified as: creatinine, CRP, serum levels of 25D, total calcium; mortality during hospitalization; the number of patients admitted to ICU or required mechanical ventilation and the duration of mechanical ventilation. 25D were assessed by CLIA.

The week long supplementation significantly increased the mean serum levels of 25D from 20.9 ng/mL to 44.4 ng/mL but did not significantly reduce hospitalization length. Among the patients with 25D deficiency at baseline, no significant differences were observed in the median hospital length of stay between the D3 and placebo group, which was in sharp contrast to #17.

#17 (49): Between April 4 of 2020 to April 22 of 2021, the multicentre international COVID-VIT-D program (NCT04552951) enrolled 548 moderate–severe COVID-19 patients from four countries (Argentina, Chile, Guatemala and Spain) and gave the treatment group an oral bolus of 100,000 IU of D3 at hospital admission beside standard care. Criteria for hospitalization included lung radiological evidence of characteristic COVID-19 disease (e.g., bilateral multifocal ground-glass opacities >50%), and/or moderate–severe flu-like symptoms (e.g., having oxygen saturation lower than 94%), and/or comorbidity. 83.1% of the admitted patients had pulmonary involvement. The most frequent symptoms were fever (71.5%), cough (66.5%), weakness (62.2%), dyspnoea (54.0%) and headache (34.6%); and the most frequent comorbidities were hypertension (43.8%), diabetes (24.7%) and cardiovascular disease (21.2%). Serum D3 were measured at the time of hospital admission locally in each center by ECLIA or CLIA, and differences of baseline serum D3 by countries were observed (median ng/mL; Argentina 16.0; Chile 19.5; Guatemala 24.1; Spain 13.4).

Three outcomes were set as the end points of the COVID-VIT-D trial: length of hospitalization, admission to the ICU and mortality. Although the supplementation raised their serum 25D from 17.0 to 29.0 ng/mL (at discharge), slightly shy from the optimal level (>30 ng/mL), it did not improve the COVID-19 outcomes. Interestingly, those with relatively high baseline serum D3 level (>25 ng/mL) were associated with a lower risk of pulmonary involvement and ICU admission, and less days of hospitalization, comparing to those with low level (≤10 ng/mL).

#18 (50): Aiming to evaluate whether VD supplementation could prevent respiratory worsening among hospitalized patients with COVID-19, this multicentre CARED trial (NCT04411446) enrolled 218 mild-to-moderate COVID-19 patients in Argentina between August 2020 and June 2021, and gave the intervention group a single high oral dose (500,000 IU) of D3 as soon as possible after randomization.

There were no significant differences between treatment groups in baseline characteristics. The primary outcome was set as change in the respiratory SOFA score between baseline and the highest value recorded up to day 7; and three secondary outcomes were ICU admission, the length of hospital stay, and in-hospital mortality. Values of ratios SpO2/FiO2 were used to calculate the SOFA scores. Risk factors included: hypertension (43.1%, *n* = 94), obesity (39.9%, *n* = 87), diabetes (26.6%, *n* = 58), chronic respiratory disease (11.9%, *n* = 26), and cardiovascular disease (4.6%, *n* = 10).

The serum 25D concentrations increased from 32.5 ng/mL at baseline to 102.0 ng/mL (7 d after supplementation) but did not prevent the respiratory worsening and had no significant effects on the length of hospital stay or other outcomes.

In the first stage, the study aimed to assess the effects of VD on SOFA, and the second stage aimed to evaluate the effects of VD on clinical events. Enrolled patients were admitted to general wards within the last 24 h, with SARS-CoV-2 confirmed infection by RT-PCR, an expected hospitalization for at least 24 h, oxygen saturation ≥ 90% (measured by pulse oximetry breathing ambient air), and at least one of the following conditions [age 45 or older or asthma (at least moderate), body mass index ≥30, chronic obstructive pulmonary disease or, cardiovascular disease (history of myocardial infarction, percutaneous transluminal coronary angioplasty, coronary artery bypass grafting or valve replacement surgery), diabetes, or hypertension]. During the first 7 d blood pressure, heart and respiratory rate, inspired fraction of oxygen, SpO2, temperature, and clinical and adverse events were recorded. Serum 25D levels were determined quantitatively by CLIA in a central laboratory.

The Steering Committee decided to stop the recruitment and terminate the trial on 7th July 2021 based on that the differences between groups, either on the primary outcome (i.e., the change in SOFA) and the secondary outcomes, did not meet the prespecified criteria to proceed to the second stage.

#19 (51): The study (NCT05166005) lasted from 30 Nov. 2020 to 20 March 2021. Two doses of bolus D3 supplementation (50,000 IU on the 1st and the 8th d of hospitalization) were given to hospitalized COVID-19 patients in Russia. The COVID-19 diagnosis was confirmed by PCR-test and/or chest CT scan, and the disease severity (mild, moderate, severe) was judged accordingly. Serum 25D levels were measured using a CLIA on microparticles. Of note, the control patients were significantly younger than VD group patients (*p* = 0.03), otherwise they were comparable and had no significant differences in baseline parameters.

The primary outcomes were set as changes of the following parameters between the first d and 9th d of hospitalization: serum 25D and CRP levels, complete blood count and B cell subsets. The secondary endpoints were set to evaluate the effects of D3 supplementation on ICU admission rates, and clinical outcomes (disease severity, hospitalization duration and oxygen supplementation).

The supplementation increased the serum 25D level by 40.7% [still shy from the recommended 25D level (40 to 60 ng/mL)], and the level on the 9th d was found negatively associated with the number of bed d (r = −0.23, *p* = 0.006), but no other differences (ICU admission rates, mortality, and the average time of hospital stay) were found between the supplemented and control groups. Immunologically, the supplementation group had significantly higher counts of neutrophil (*p* = 0.04), lymphocyte (*p* = 0.02), and CD27^−^CD38^−^ double negative B cells (*p* = 0.02), but lower CRP (*p* = 0.02 at the 9th d of hospitalization) and frequencies of CD38^++^CD27 transitional and CD27^−^CD38^+^ mature naive B cells (*p* = 0.006 and *p* = 0.02). Thus, VD supplementation raised 25D level and affected immunity, which might contribute to change their course of COVID-19 in VD insufficient patients.

#20 (52): After sample size was calculated using Package “Medcalc” (trial version) and random allocation sequence generated using Microsoft Excel, 117 Tunisian patients (mean, 42 y; 65.8% of the participants were asymptomatic) who remained RT-PCR positive for COVID-19 on the 14th d were enrolled in the trial (NCT04883203), and 57 of them received a single dose of 200,000 IU of D3. The intervention was made by medical residents, starting from May to August 2020. The primary outcome was set as the recovery delay (defined as the period between the day of the 14th RT-PCR-positive result and the day of the second successive negative RT-PCR test result), and secondary outcomes were set to monitor the changes of SARS-CoV-2 RT-PCR cycle threshold values between that at the beginning (date of randomization) and the second successive negative RT-PCR test.

Compared to the placebo group, this bolus D3 supplementation had not shortened the recovery delay. Conversely, the median duration of RNA viral conversion was significantly longer in the D3 supplementation group than in the placebo group. One of the limitations of this study, as the authors admitted, is that 25D serum levels was not measured, neither at enrollment nor after VD supplementation.

#21 (53): COVID-19 PCR positive patients needing invasive or non-invasive respiratory support were eligible for inclusion in the trial (NCT05384574), and 155 severe (on respiratory support) COVID-19 patients admitted to ICU in a Croatian hospital were enrolled and randomized using a computer-generated code to receive D3 supplementation (10,000 IU daily for 14 d). All patients included in this study received standard care. Mechanical ventilation was applied with protective lung ventilation using tidal volumes 4–8 mL/kg and plato pressures ≤30 cm H_2_O.

Dexamethasone was routinely administered to all eligible patients. Number of days spent on respiratory support (invasive or non-invasive) was set as the primary outcome; secondary outcomes included: all-cause mortality on d 14, 28 and 60, clinical improvement at d 28 (according to WHO clinical progression scale), number of days spent in ICU, number of days spent in hospital, bacterial superinfections, neutrophil to lymphocyte ratio and disease severity (CRP levels, PaO_2_/FiO_2_ ratio, D-dimer levels, fibrinogen, ferritin, PCT).

All these patients had vitamin D levels measured on admission. VD supplementation began within 48 h of admission to ICU and last for at least 14 d during ICU stay or anywhere else orally or via gastric tube by experienced nursing staff. Disease severity markers were collected daily during ICU stay and every third d after discharge from ICU. VD levels were checked three times (on admission to the ICU, d 7 and 14; of note, VD levels were not measured for patients in the control group on d 7 and 14), and measured in a hospital laboratory using the ECLIA method.

The mean 25D level before supplementation (on admission) was 27.1 nmol/L (10.8 ng/mL), which reached 38.5 nmol/L (15.4 ng/mL) and 56.2 nmol/L (22.5 ng/mL), respectively, on d 7 and 14 after supplementation. The supplementation had not made a difference in either the main (days on respiratory support) or any of the secondary outcomes (days spent in ICU or length of hospital stay). The sample size was shy from the calculated one to detect a 2-day difference in number of days on respiratory support (137 patients in each group) due to short of patients admitted.

#22 (54): A total 106 hospitalized Iranian patients who had a circulating 25D concentration of <30 ng/mL and in need of respiratory support were enrolled in this multicenter study registered at https://ClinicalTrials.gov with the identifier number blinded in the journal article. The patients presented acute respiratory tract infection symptoms (eg., cough, dyspnea, fever) and the COVID-19 diagnosis was confirmed by RT-PCR and/or chest CT scan findings compatible with COVID-19. The participants were randomly grouped (no significant age and sex differences) in their first visit and received either a bottle containing 30 capsules of 25D or placebo. The same standard care (a combination of hydroxychloroquine and azithromycin) were given to all patients, and ceftriaxone for patients with pneumonia.

The study outlined six outcomes: 1, percentage of COVID-19 severity (mild, moderate, and severe) based on WHO criteria; 2, length of hospital stay counting from admission to discharge; 3, percentage of patients who need oxygen support; 4, rate of death due to COVID-19; 5, lymphocyte count and percentage; 6, serum 25D concentrations at three time points (baseline, 30 and 60 d after enrollment).

After supplementation, the circulating 25D concentrations significantly increased (30 d, 42.0 ng/mL; 60 d, 59.6 ng/mL; placebo 19.3–19.4 ng/mL), meeting one of the six outcomes outlined for the trial. However, there was no statistically significant difference in four of the other (clinical) outcomes (ICU admissions, need for ventilation, length of stay and rate of death) between the two groups.

#23: Hospitalization took a COVID-19 patient seven d (median) counting from the symptom onset, and most (85.9%) of the enrolled 85 Spanish severe COVID-19 patients showed bilateral pneumonia on x-rays. The main coexisting comorbidities were obesity (54.1%), hypertension (48.2%), dyslipidemia (36.5%), and diabetes (22.3%). The primary endpoint was the increase of 25D serum level ≥ 30 ng/mL after 14 d of the end of supplementation.

After D3 supplementation for 2 weeks in combination with the standard care in patients hospitalized with pneumonia due to COVID-19, the mean serum 25D levels increased from 14.8 ng/mL to 19.11 ng/mL in the low dose group (2000 IU/day), and to 29.22 ng/mL in the high dose group (10,000 IU/day) (*p* < 0.0001), which was still slightly shy from the primary endpoint (25D serum level ≥ 30 ng/mL). The length of hospital stay (one of the secondary endpoints) was not significantly different between both groups. However, beneficial effect (shorter stay at the hospital) was only observed in participants who developed ARDS and received high dose D3 supplementation when compared to the low dose group (8.0 vs. 29.2 d, *p* = 0.0381). The levels of haemoglobin and bilirubin in the high VD (10,000 IU/d) group were significantly higher (*p* = 0.006 and *p* = 0.010, respectively) at the end of supplementation, compared to low VD group.

#24 (55): 90 moderate to severe COVID-19 patients (45 each group) who were VD deficient were enrolled in this trial (SHADE-S, NCT04952857) to test the effect of high oral dose of D3 supplementation on SARS-CoV-2 clearance. The patients (13–14 d from symptom onset to recruitment) presented CT scan findings of the lung (bilateral multifocal ground-glass opacities ≥50%) and PaO2 /FIO2 lower than 200, an indication requiring invasive/non-invasive ventilation. The SARS-CoV-2 infection diagnoses were further confirmed by RT-qPCR. Upon admission at breakfast, patients received orally either placebo (medium-chain triglyceride oil) or a single high dose (0.6 million IU) of D3 (nano-droplet forms).

The primary outcome was the difference in SOFA score at d 7 between the two groups, and the secondary outcomes measured change in SOFA scores (at D 3, 10, 14), PaO2/FiO2 ratio, total duration of mechanical ventilation, all-cause mortality within 28 d of intervention, and the change in inflammatory markers (CRP, d-dimer, ferritin). The 25D levels were measured by in-house ECLIA using a kit. VD deficiency was defined as 25D level < 20 ng/mL, and severe VD deficiency <10 ng/mL.

Seven d after, the median 25D levels of the VD group raised from 12 ng/mL at baseline to 60 ng/mL whereas the placebo group changed from 13 ng/mL to 16 ng/mL; the SOFA score and PaO2 /FiO2 ratio of the VD group significantly improved (*p* = 0.01). D3 supplementation also significantly lowered CRP (*p* = 0.003) by d 7 and all-cause mortality at d 28 (*p* = 0.046); and interestingly a decrease in total calcium over time. Days on mechanical ventilation were lower in the VD group.

#25 (56): 50 patients were allocated through electronic randomization on the day of admission. They received no treatment or with calcitriol (1,25D) 0.5 μg daily for 14 d or hospital discharge (whichever was first). Four outcomes were prespecified: length of hospital stay, need for ICU admission, mortality, oxygen requirements, and readmission. 25D levels were not measured. Only a significant reduction in oxygen requirements in those who were supplemented.

## Summary of, comment to and discussion on the 25 RCTs

### Short summaries of the 25 RCTs

The 25 RCTs enrolled COVID-19 patients of diverging severity by local (regional or national) criteria, ranging from asymptomatic or mild (35), mild to moderate (36, 42, 54), moderate-to-severe (41, 48), severe (53, 57), to pneumonia (34, 43).

D3 (cholecalciferol) is the most often used form of supplemental VD (58), which is also the case in our reviewed RCTs (Table 1). 20 of the 25 RCTs supplemented with D3 manufactured by different companies, and the others used special VD supplements: two with 25D (34, 54), three with alphacalcidol [(41, 43, 56); the lower dose arm of RCT#8 used alphacalcidol too]. Comparing to its precursor D3, 25D supplementation is direct (no need to be converted in the liver) and more hydrophilic, absorbed better in the intestine (79% vs. 93%), therefore 3.2-fold more potent [calculated and reviewed in Quesada-Gomez and Bouillon (59)]. Conversely, 1 μg of D3 increased serum 25D by 1.5 ± 0.9 nmoL/L whereas 25D increased it by 4.8 ± 1.2 nmoL/L (59). Alphacalcidol (1α-D3) is a non-endogenous VD analogue, which is structurally different from D3 (Figure 1), and has longer pharmacological actions than D3 because of a negative feedback mechanism regulating the final activation step in the kidneys (60).

**Table 1:** Some features of the reviewed RCTs.

RCT	Participants		Mean age (years)		VD type	Dose (IU) (cumulative dose)	How/when	How long	VD level (ng/ml, some changed from nmol/L)		Country	PMID (ref)
	VD	C/P	VD	C/P					pre	after		
#1	50	26	53	52.8	25D	532 mcg/d 1, 3, 7; then 266 mcg /w	See left box	UD or ICU	NA	NA	Spain	32871238 (34)
#2	16	24	50	47.5	D3	60,000 (4.8E5 to 8.2E5)	Daily/1 w; weekly	1 or 2 w	D 8.6 P 9.5	D **51.7** P 15.2	India	33184146 (35)
#3	36/33	0	46.3/53.5	NA	D3	1,000 or 5,000 (1.4E4 or 7E4)	Daily	2 w	H 21.4 L 25.2	H 25 L 21.9	Saudi Arabia	34202578 (36)
#4	161	160	36	39	D3	4,000 (1.2E5)	Daily	1 m	D 18.4 P 16.5	D 26.1 P 19.3	Mexico	35487792 (37)
#5	127/127	0	87/89	NA	D3	50,000 or 400,000 (5E4 or 4E5)	**Once** only	NA	H 20.8 S 17.2	H **60.4** S 25.6	Netherlands	35639792 (38)
#6	26	24	63.2	68.7	D3	25,000 d: 1–4, 8, 15, 22, 29 (2E5)	See left box	8 d/m	D 17.9 P 16.9	D 29.9 P 16.9	Belgium	35893907 (39)
#7	20	25	10.7	14	D3	1,000 or 2,000 (1.4E4 or 2.8E4)	Daily	2 w	D 13.8 C 11.4	NA NA	Mexico	35958172 (40)
#8	58/58	0	66.1/65.7	NA	1 − α 25D/D3	25D: 1mcg/ d or D3:200,000 (**IM**) (2E5)	25D: daily; D3: **once**	5 d	NA	NA	Egypt	36295519 (41)
#9	65	69	42.1	43.8	ERC	300 mcg /d 1–3 & 60 mcg /d 4–27	Daily	27 d	D **37.7** P **37.1**	D **81.8** P **34.8**	USA	36529089 (42)
#10	147	147	47.9	53.7	1 − α 25D	2 mcg	Daily	UD	D 22.5 C 20.8	NA NA	Thailand	38383361 (43)
#11	75	162	63.3	63.3	D3	5,000 (7E4)	Daily	2 w	9.64	NA NA	Austria	34976511 (44)
#12	25	25	41.4	46.4	D3	360 (0.5E4)	Daily	2 w	NA	NA	Pakistan	35747751 (45)
#13	30	30	50	26.0	D3	2,000 (2.8E4)	Daily	2 w	NA	NA	Egypt	36425571 (46)
#14	90	91	NA	NA	D3	180,000, then 2,000/d (2.92E5)	**Once**, then daily	8 w	D 18.2 P 23.5	D 26.7 P 22	India	37560461 (47)
#15	1,550 1,550	3,100	?	60.8	D3	800 or 3,200 (1.44E5 or 5.76E5)	Daily	6 m	H 16.4 L 16.6 C NA	H **41.2** L **31.**6 C 21.5	Brazil	36215226 (61)
#16	120	120	/	/	D3	200,000 (2E5)	**Once** only	NA	D 21.2 P 20.6	D **44.4** P 19.8	Brazil	33595634 (48)
#17	274	269	59.0	57.0	D3	100,000 (1E5)	**Once** only	NA	D 17 C 16.1	D 29 C 16.4	Spain	35177066 (49)
#18	115	105	59.8	58.3	D3	500,000 (5E5)	**Once** only	NA	D **32.5** P **30.5**	D **102** P **30**.0	Argentina	35622854 (50)
#19	56	54	58	64	D3	50,000 (5E4)	d1 d8	NA	D 16.4 P 13.9	D 22.8 P 10.6	Russia	35807783 (51)
#20	57	60	43	41	D3	200,000 (2E5)	**Once** only	NA	NA	NA	Tunisia	36803273 (52)
#21	75	77	65	65.5	D3	10,000 (1E4)	Daily	≧2 w	D 10.1 C 10.9	D 22.5 NA	Croatia	36904232 (53)
#22	53	53	50	49	25D	25 mcg	Daily	2 m	D 19 P 18	D **59.6** P 19.4	Iran	34653608 (54)
#23	41/44	0	67/65.3	NA	D3	2,000 or 1,0000 (2.8E4 or 1E5)	Daily	2 w	H 15.3 L 14.3	H 29 L 19	Spain	35468580 (57)
#24	45	45	51	46	D3	600,000 (6E5)	**Once** only	NA	D 12 P 13	D **60** P 4	India	38291897 (55)
#25	25	25	69	64	1,25D	0.5 mcg	Daily	2w	NA	NA	USA	34508882 (56)

25D, 25-hydroxycholecalciferol, calcifediol; c, control; C/P, Control/Placebo; d, day; (V)D, vitamin D; D3, cholecalciferol, calcidiol; ERC, extended-release calcifediol; H, high dose; IM, intramuscular injection; L, low dose; m, month; MCG, microgram; NA, not available/applicable; once (only), a single dose; p, placebo; RCT,; S, standard dose; w, week(ly); y, year; UD, until discharge; ICU, ICU admission; numbers after one decimal are rounded up; 1E4 = 10,000; 10 mcg of calcifediol = 2000 IU of D3 https://vitamored.com/products/vitamored-vegan-vitamind3-calcifediol. Bold values indicating above the sufficiency level.

Regarding the dose and dosing strategy, there were three patterns. Over half of them (14/25) used ‘same daily dose’ for some time, ranging from a shortest period of 5 days (41) to the longest of 6 month (61); nine of them either used ‘two high doses (RCT#19, each on day 1 and day 8)’ or a single high dose (9 RCTs, #: 5, 8, 16–21 and 24); and three used ‘intermittent higher to lower doses’ (RCT#: 1, 6 and 14).

It was reported that high-dose bolus replacement may induce long-term expression of the catabolic enzyme 24-hydroxylase and fibroblast growth factor 23, both having VD inactivating effects (62), which might be one of the reasons why high-dose bolus of VD in some trial did not work (51, 52). Contrarily, a 3-month long RCT study comparing efficacy of daily, weekly and monthly administration of D3 demonstrated equal efficacy (63).

For ethical reasons, some RCTs had not set up placebo group (eight RCTs, #: 1, 7, 10, 12, 13, 17, 19, and 21) or lacked both control/placebo groups (six RCTs, #: 3–5, 8, 11, and 23). Some were single-blinded (RCT#: 23 and 25) or double blinded (seven RCTs, #: 1, 4, 14, 16, 18, 22, 24), and some were open-labeled (nine RCTs, #: 1, 5, 10, 11, 15, 17, 19, 21, and 26).

Among the trials with 25D serum concentration data available, only two RCTs had optimal (≥30 ng/mL) levels at baseline (42, 50), the others were in VD deficit status (ranging between insufficiency to severely deficiency). After supplementation by varying strategies, ten RCTs had endline 25D serum concentrations slightly shy from [De Niet et al. (39), Cannata-Andía et al. (49), and Torres et al. (57)] or beyond the optimal level (35, 38, 42, 48, 50, 54, 61).

### Comments to and discussion on the 25 RCTs

Considering the nearly hopeless situation the COVID-19 pandemic had put the whole world in, it is understandable that the 25 RCTs we here focused on had set various and sometimes very different prespecified primary and secondary outcomes.

Judging from a clinical perspective, one would say some RCTs in this series of trials fully or partly succeeded (e.g., RCTs #1–6, 8–10, 23, 24), and some failed (RCTs #17–22). Following are a brief comment to and comparison of some of the trials and, discussion of the possible reasons of their success and failure.

No matter successful or not, some RCTs lacked a key information, the 25D levels at baseline and/or after supplementation of some/all groups, which hinders comparison analysis between baseline and endline VD status (sufficient, insufficient, or deficient) of the tested subjects that is one of the central points of VD supplementation. That is the case for eleven out of the 25 trials (detailed in Table 1); specifically, six RCTs (#: 1, 8, 12, 13, 20, 25) lacked the full set data (25D values at baseline and after supplementation), and five RCTs (#: 6, 7, 10, 11, 21) had incomplete information. So, the discussion will basically leave some of these eleven RCTs out due to lack of data.

One thing to bear in mind is that trial success is relatively speaking and also related to the severity of the infected participants, it will be hard to imagine the outcome if RCT#2 and RCT#3 enrolled severe COVID patients instead of mild to moderate COVID-19 patients (35, 36).

Technically speaking, RCT#2 succeeded in meeting its prespecified outcome (35). Compared to placebo group, more people in the supplementation group became SARS-CoV-2 RNA negative (*p* < 0.018), in contrast to the failure of RCT#20 in this aspect (52). The main difference in supplementing D3 to boost the VD level is that RCT#20 used a single high dose (200,000 IU) without knowing baseline and endline VD status, while RCT#2 used 60,000 IU daily per oral to make the 25D level reach >50 ng/mL at day 7; if not, continued for another week. The supplemented patients in RCT#2 received at least twice the total dose compared to the single high dose only in RCT#20. Of note, RNA test from positive to negative only indicates the decrease of the potential contagiousness, not exactly equal to clinical improvement(s).

RCT#3, together with the other five trials (RCTs #5, 16–18, 22) were carried out at multiple centers. Two met (RCT# 3 and 5) but the rest three failed to meet their respective outcome(s). There are several interesting with some baffling findings from these six multicenter trials. As of VD types for supplementation, only RCT#22 used daily 25D for 30–60 d (54), and the other five used D3 by differing strategies. Strategy-wise for the five trials using D3, RCT#3 used daily 1,000 or 5,000 IU for 2 weeks (36) while the others used single high doses: 50,000 or 400,000 IU in RCT#5, 100,000 IU, 20,0000 IU, or 500,000 IU, respectively, for RCTs #17, #16 and #18.

Comparing to failure of their respective low dose group (1,000 IU daily for 2 weeks and 50,000 single dose) in RCT#3 and RCT#5, both of their high dose groups (5,000 IU daily for 2 weeks or 400,000 IU single dose) succeeded to achieve their respective clinical outcomes. Comparing to the high dose groups of RCT#3 and RCT#5 and their endline mean 25D levels (25 ng/mL vs. 60 ng/mL), the other four trials using either single high dose or daily VD achieved comparable or even higher endline mean 25D levels (44.4 ng/mL in RCT#16, 29 ng/mL in RCT#17, 102 ng/mL in RCT#18, and 42 ng/mL in RCT#22). The baffling question is why the other four trials failed to meet their respective outcomes while they succeeded to significantly correct the VD level? There may be other rational hypotheses, but one of the possibilities is that the endline 25D serum concentration upon VD supplementation is not an indicator guaranteeing success in counteracting a COVID-19 infection.

There were several differences among these six trials besides differences of dose, dosing strategy and serum 25D levels. They enrolled COVID-19 patients of different features: mild to moderate severity of patients in RCT#3 and RCT#16–18; while the high dose group in RCT#3 was younger and less obese (the outcome difference remains significant after adjusting for age, sex, baseline BMI, and D-dimer); RCT#16 had several nationalities with varying baseline 25D levels (ng/mL; Guatemala 24.1, Chile 19.5, Argentina 16.0, Spain 13.4); more than half were white and over 30% mixed ethnicity in RCT#17 while it took 10 days from symptom onset to enrollment; and patients in RCT#18 had risk factors for disease progression; RCT#5 had old patients (median age 88) who was diagnosed by RT-PCR or chest CT scan within 72 h in (also the latest time VD supplementation started); and the severity of patients in RCT#22 was not specified and the trial was terminated after reviewing of first stage results. All the above distinct baseline characteristics can theoretically affect the outcomes. Genetic factors influence not only baseline serum 25D concentration (64), but also the response to VD supplementation (65). Similarly, some other RCTs also had not specified the criteria of defining the severity of the infection (23, 35, 41, 43, 44, 47, 49, 51, 52), some (34, 46) used well defined criteria with a traceable reference [such as (66)], the CURB65 severity scale (67), and some employed criteria self-defined (42, 45, 48, 50) or outlined by their local health departments (36, 40). For example, corticosteroids, used in some RCTs (41, 43), could confound the outcomes of VD supplementation on COVID-19 infection (68).

Moreover, serum 25D was measured differently, which is also a key concern expressed in a recent review (58). RCT#3 used a fully automated CLIA analyzer, RCT#5, RCT#16 and RCT#18 used CLIA, RCT#17 used ELIA or CLIA, and RCT#22 used HPLC (54). The differences between different assays are huge, and even the same type assay but using different protocols and/or reagents, which all contribute to variations of the serum 25D values.

Two RCTs were aimed to investigate whether VD supplementation could prevent COVID-19. RCT#4 was successful in significantly lowering SARS-CoV-2 infection rate in frontline healthcare workers (37). By contrast, RCT#15 concluded that VD supplementation was unable to reduce the risk of COVID-19 (61). Here the 25D puzzle came again. The ‘4,000 IU VD daily for 30 d’ strategy in RCT#4 had changed the 25D level from 18.4 ng/mL to 26.1 ng/mL (below the sufficient level) but it worked to lower the COVID-19 (37). On the contrary, daily supplementation of VD (800 IU or 3,200 IU) for 6 month raised the VD level from about 16 ng/mL to 31.6 ng/mL and 41.2 ng/mL (61), both above the sufficient level set by the Society (19), but both failed to reduce risk of COVID-19.

Besides the dose/dosing difference, there were other differences between these two trials. RCT#4 was a relatively small scale trial, with less than 200 Mexican healthcare workers (median age 36.5), and some of them might unknowingly have exposed to COVID-19 due to their occupation risk, become immunized but remained asymptomatic, because only RT-PCR tests were taken at baseline (37). Moreover, the VD group was significantly younger (36 vs. 39, *p* = 0.019); the concentration of 25D was determined using a Waters ACQUITYH UPLC. RCT#15 was the largest among all trials, with more than 6,000 participants of median age of 60.2 and about 95% were white (61). Of note, concentrations of both serum 25D (25D_3_ and 25D_2_) were measured by LC-TMS using dried blood spot eluates (61).

RCT#6 used an intermittent dosing strategy (25,000 IU per day for the first 4 d, then the same dose per week for up to 6 weeks) and raised the serum 25D from 17.9 to 29.9 ng/mL, slightly shy from the sufficient level. The supplementation decreased significantly the length of hospital stay (39).

RCT#7 was the only one targeting pediatric COVID-19 patients, but it was prematurely ended due to ethical reason (40), and apparently such trial is warranted in the future. Although still controversial, the general consensus from studies of different natures (sectional, observational, cohort) suggests VD supplementation is essential to those whose VD levels are sub-optimal but the usage should be carefully monitored to prevent overdosing (58).

RCT#8 have two interesting features. One is that in the same trial the investigators decided to use two different VD supplements by two different routes for supplementation. The low dose group orally took 25D (alfacalcidol; 1 mcg/day) for 5 days, and the high dose group received an extra single intramuscular injection of D3 (cholecalciferol; 200,000 IU) upon enrollment. The other interesting feature is that the investigators knew the virus causing these infections was a D614G mutant strain which was prevalent in Egypt at the trial period and associated with higher viral loads and probably with enhanced transmissibility compared to other variants (66). Among the 25 RCTs reviewed, this is the only study reported the causal virus information of the trial. It is well-known that SARS-CoV-2 variants differ in their virulence and epidemiology, causing COVID-19 diseases of varying severity, which is also relating to evaluation of therapy and prevention trials. Unfortunately, it lacked serum 25D data both at the baseline and after supplementation (41), which made it impossible to compare their VD correction effects of the different supplementation routes (oral vs. intramuscular) and types of VD supplements used (D3 vs. 25D).

RCT#9 has two unique features among all. It is the only trial using extended-release calcifediol (ERC) for supplementation, and one of the only two trials that enrolled participants whose baseline VD were at the sufficient levels (42, 50). ERC has a different pharmacokinetics than the conventional 25D product (used in RCT#22) by releasing 25D gradually over a period of 12 h (69).

RCT#10 demonstrated that VD supplementation only worked for a subset of patients (those require supplemental oxygen or high-dose corticosteroid therapy or have high CRP >30 mg/L), but not to all enrolled patients, which helps understand the conflicting results between all trials (43).

RCTs #11–14 were trials using VD as one of the combo components, they were either designed without an VD alone group or VD alone did not work at all (44–47). In the future, similar of such combo trials need better designed to have a VD alone group if wanting to see the independent effect(s) of VD supplementation.

RCT#24 and RCT#2 were the only two RCTs carried out in the same hospital (Nehru hospital of north India) among the 25 trials reviewed. RCT#2 (SHADE; NCT04459247) started 1 year earlier (2020-06-15) than RCT#24 (SHADE-S; NCT04952857; 2021-08-01). One of the difficulties when comparing single center RCTs is that they were carried out in different medical institutions using different brands of similar machines, reagents, and protocols to run assays, which creates heterogeneous results. In this sense, it is interesting to compare these two trials held in the same institution using very similar if not identical logistics (machines, reagents, and protocols). The only major differences between these two trials would be on the patients and the dose/dosing. Still, there are similarities and differences between them.

Similarities. Both trials were successful and of the same design (randomized, double-blind, placebo-controlled) and comparable scale (less than 100 patients), enrolled vitamin D-deficient patients, used the similar if not identical instruments, reagents and protocols to measure 25D, inflammatory markers and so on (35, 55).

Major differences. The SHADE study enrolled asymptomatic or mildly symptomatic patients (thus, different baseline values of their demographic parameters, inflammatory markers and so on), gave the intervention group daily 60,000 IU of D3 for 7 d (or 14 d if 25D not >50 ng/mL), looked for SARS-CoV-2 RNA negativity as the primary outcome (35). By contrast, the SHADE-S trial enrolled severe COVID-19 patients and gave the intervention group a single high-dose (0.6 million IU) of D3, and looked for SOFA score at D 7 as the primary outcome (55).

## Conclusion

Together, the general conclusion from these studies is that VD insufficiency/deficiency is highly related to COVID-19 infection, its severity and mortality, but data of the effect on clinical benefit from VD supplementation is conflicting, further RCT study is surely needed.

One key but puzzling observation after carefully reviewing these 25 RCTs is that the endline serum 25D concentration, although a good indicator of the VD supplementation effect on correcting VD insufficiency/deficiency, it is not reliable to predict that VD sufficiency after supplementation is a guarantee of clinical improvement of COVID-19. There are 7 RCTs reviewed above that had endline serum 25D concentration at or above the optimal level (#: 2, 5, 9, 15, 16, 18, 22; the exact ng/mL values in Table 1; optimal level ≥ 30 ng/mL) but only three reached its trial outcome (RCTs, #2, 5, 9) and the other four failed, despite having the VD deficit of the patients corrected. By contrast, the supplementation in three RCTs (#3, 4, 6) failed to correct the VD insufficiency/deficiency but succeeded in improving the clinical outcome(s). Apparently, the scientific community need to work out a (set of) biomarker(s) that can be used as a correlate of the effect of VD supplementation on protection (prophylactic), treatment (therapeutic) or both.

Although it is a long way to go, there are already some pioneering work that has been done. Among others, calprotectin (70, 71), endocan (72), growth differentiation factor 15 (GDF15) (73), inflammatory cytokines (IL1 and IL6) (74), miRNAs (75), neopterin (76), soluble suppressor of tumorigenicity 2 (sST2) (77), and T cell immunoglobulin and mucin domain containing protein 3 (Tim) (78) have been reviewed having the potential as biomarkers for COVID-19 severity.
